# Tool Path Design of the Counter Single Point Incremental Forming Process to Decrease Shape Error

**DOI:** 10.3390/ma13214719

**Published:** 2020-10-22

**Authors:** Kyu-Seok Jung, Jae-Hyeong Yu, Wan-Jin Chung, Chang-Whan Lee

**Affiliations:** Department of Mechanical Design and Manufacturing Engineering, Seoul National University of Science and Technology, Seoul 01811, Korea; ksjung@seoultech.ac.kr (K.-S.J.); jh9109@seoultech.ac.kr (J.-H.Y.); wjchung@seoultech.ac.kr (W.-J.C.)

**Keywords:** incremental sheet metal forming process, compensation, counter forming, tool path

## Abstract

Incremental sheet metal forming can manufacture various sheet metal products without a dedicated punch and die set. In this study, we developed a two-stage incremental forming process to decrease shape errors in the conventional incremental forming process. The forming process was classified into the first single point incremental forming (1st SPIF) process for forming a product and the counter single point incremental forming (counter SPIF) process to decrease shape error. The counter SPIF gives bending deformation in the opposite direction. Furthermore, the counter SPIF compensates for shape errors, such as section deflection, skirt spring-back, final forming height, and round. The tool path of the counter SPIF has been optimized through a relatively simple optimization method by modifying the tool path of the previous step. The tool path of the 1st SPIF depends on the geometry of the product. An experiment was performed to form a circular cup shape to verify the proposed tool path of the 1st and counter SPIF. The result confirmed that the shape error decreased when compared to the conventional SPIF. For the application, the ship-hull geometry was adopted. Experimental results demonstrated the feasibility of the two-stage incremental forming process.

## 1. Introduction

In order to meet the customer’s various needs, a flexible manufacturing system, which includes flexibility for small batch production [1], is increasing. Incremental sheet metal forming is a suitable forming technology for the aforementioned types of flexible manufacturing systems [2]. This corresponds to forming technology that produces the desired shape by increasing small deformations in contact with the tool and sheet.

**Figure 1:**
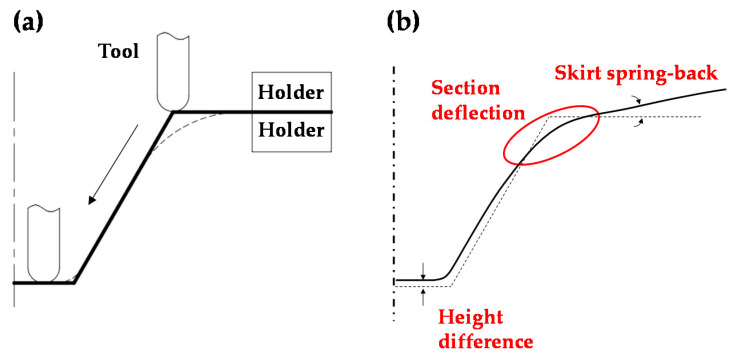
(**a**) Single point incremental forming process. (**b**) Shape errors in the single point incremental forming (SPIF) process.

Iseki et al. [3] proposed single point incremental forming (SPIF). The incremental forming technology uses only a three-dimensional tool path and a simple forming tool on a CNC machine. Typical sheet metal forming requires the production of a die and punch. However, the incremental forming employs a round tool and tool path, as shown in Figure 1a, instead of a die-set. Consequently, the formability exceeds that of a typical forming technology, and thus various products can be manufactured [4]. 

However, shape errors occur during and after forming in incremental forming [5]. During forming, bending deformation occurs in the sheet by the force of the tool pressing the sheet and the force of the blank holder holding the sheet [6]. Furthermore, after forming, skirt spring-back occurs due to the elastic recovery of the sheet. It is impossible to produce the desired shape despite the aforementioned problems. Figure 1b shows a schematic drawing of shape errors, such as the section deflection, spring-back, and height error in the formed shape.

There are many studies to increase formability and accuracy in SPIF. Duflou et al. [7] employed a laser beam to heat locally where the tool contacts the material. Local heating resulted in reduced process forces, improved dimensional accuracy, and increased formability. Allwood et al. [8] adopted the closed-loop feedback control in the single point incremental forming process. The tool path was modified with the iterative process. Bambach et al. [5] increased the geometric accuracy by employing multi-stage forming. Malhotra et al. [9] calculated the proper scallop height in a 3D spiral tool path to increase geometric accuracy and decrease forming time. Zhu et al. [10] developed a 5-axis incremental forming tool and related tool paths to reduce the residual height of the product. Yang and Chen [11] studied the twist phenomena in SPIF and proposed an alternate tool path to restrain twist deformation.

Matsubara et al. [12] proposed two point incremental forming (TPIF). TPIF employs a fixture or die-set at the bottom of the sheet and the tool moves on the sheet with the designated tool path. Nguyen et al. [13] employed a negative wooden die to manufacture a small automotive white-body. Silva and Martines [14] studied the deformation mechanics of SPIF and TPIF through an analytic model and experiments. Panjwani et al. [15] introduced a flexible bolt support technique with an array of bolts in the incremental forming process. Ou et al. [16] studied process parameters of TPIF with a positive die-set to improve thickness uniformity for an irregular stepped part. 

Meier et al. [17] and Maidagan et al. [18] proposed the concept of double side incremental forming (DSIF), in which two tools simultaneously form two sides of a sheet. DSIF employs two moving tools acting as a forming tool and a supporting tool to increase flexibility and geometric accuracy. Malhotra et al. [19] and Moser et al. [20] studied forming strategies in DSIF to increase formability and geometric accuracy. Praveen et al. [21] compensated the tool paths in DSIF using an analytic model to enhance the geometric accuracy. 

**Figure 2:**
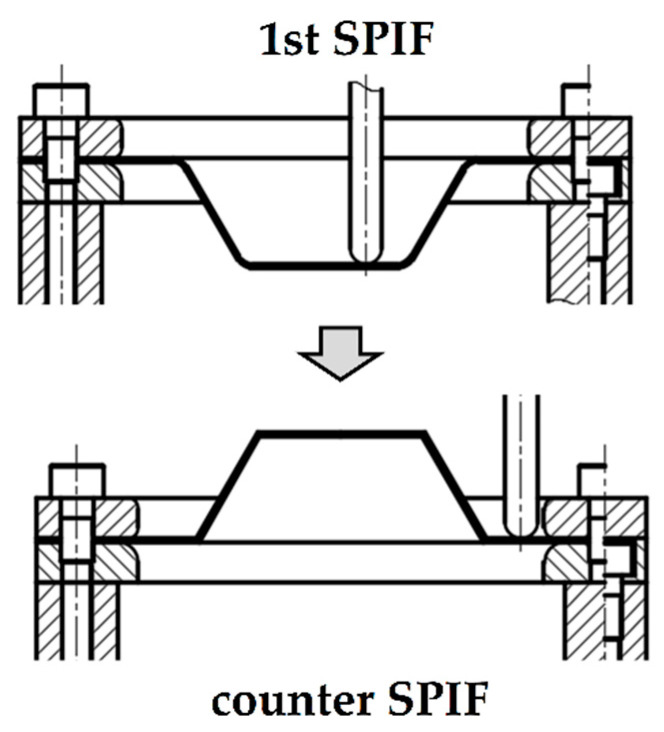
Schematic drawing of the conventional incremental forming process and the counter forming process to decrease shape error.

In this work, the two-stage SPIF process was developed to decrease shape error in SPIF. Jung et al. [22,23] proposed a new additional incremental forming process for after the conventional incremental forming process. Figure 2 shows a schematic of an incremental forming process to decrease shape errors in SPIF. The forming process rotated the sheet 180° after general incremental forming. The sheet after the first incremental forming (1st SPIF) was rotated, and the tool created deformations to reduce shape error. However, they just showed the possibilities of the proposed process. The height of the manufactured product was different from the CAD data.

There have been many studies about the finite element analysis of the incremental forming process. Said et al. [24] simulated ductile fracturing of the incremental forming process. Kim et al. [25] studied the effects of the numerical simulation conditions, such as the contact condition and element types, on the simulation results. Guzmán et al. [26] and Bouhamed et al. [27] adopted Hill’s quadratic anisotropic non-associated flow rule with mixed isotropic nonlinear kinematic hardening to simulate the deformed geometry in the incremental forming process. Thickness distribution and plastic strains were predicted property with the finite element analysis. Still, the finite element simulation of SPIF was not sufficient to predict the deformed geometry after spring-back accurately [28]. Another problem is the simulation time. The simulations of SPIF require more time than experiments. Consequently, experiments were conducted to find out the proper tool paths instead of the finite element analysis.

The first conventional incremental forming process was denoted as the single point incremental forming (1st SPIF). The additional forming process was denoted as the counter single point incremental forming (counter SPIF). In the study, we proposed a procedure to construct the tool path for counter SPIF to decrease shape error. The tool path in the counter SPIF process was modified by measuring the shape error after the 1st SPIF process. Using a simple optimization method, the tool path in counter SPIF for minimizing shape error was compensated. Additionally, the shape error along the direction, the skirt angle, and the height was corrected using the clover-shaped tool path in the counter SPIF. Experiments with the circular cup shape and the ship hull shape were performed to verify the proposed method. 

## 2. Experimental Set-Up 

### 2.1. Equipment for the Experiments

Aluminum (Al5052) was used as the material in this study. The elastic modulus, yield strength, and ultimate tensile strength were 79.5 GPa, 167.8 MPa, and 223.4 MPa, respectively [17]. The sheet size was 150 mm × 150 mm, and the thickness was 1 mm. The blank holder was machined to have a square hole with a size of 100 mm × 100 mm. Consequently, the formable size of the sheet was 100 mm × 100 mm. In this experiment, the size of the blank holder was 150 mm × 150 mm and the length of the spherical tool was 120 mm, and the radius of the spherical tool was 4 mm. 

**Figure 3:**
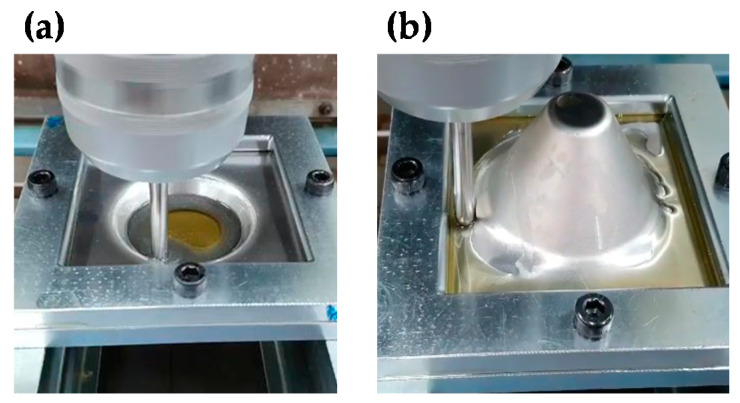
The incremental forming process of (**a**) first single point incremental forming (1st SPIF) and (**b**) counter SPIF.

A CNC machine (NAMSUNG NR-35, Seoul, Korea) was used to do the sheet in incremental forming. The CNC machine was controlled in the x, y, and z axes; the bed of the CNC machine moved in z direction and the head moved in x and y directions. Pictures of 1st SPIF and counter SPIF are presented in Figure 3. After finishing the 1st SPIF, the sheet was rotated 180°, as shown in Figure 3b, and the counter SPIF was conducted.

### 2.2. Experimental Conditions

In this experiment, the feed rate used was 2000 mm/min, and the spindle rotating speed was 200 r/min. In general incremental forming, the formability of the material and the surface quality depend on the process parameters such as feed rate and the spindle rotating speed [29]. Fracture or burr may occur without proper feed rates and spindles in incremental forming to reduce shape error. The process variable was fixed with the above values, which resulted in reasonable formability and surface quality [16].

## 3. Tool Path Design for the Counter SPIF

### 3.1. Definition of the Shape Error

**Figure 4:**
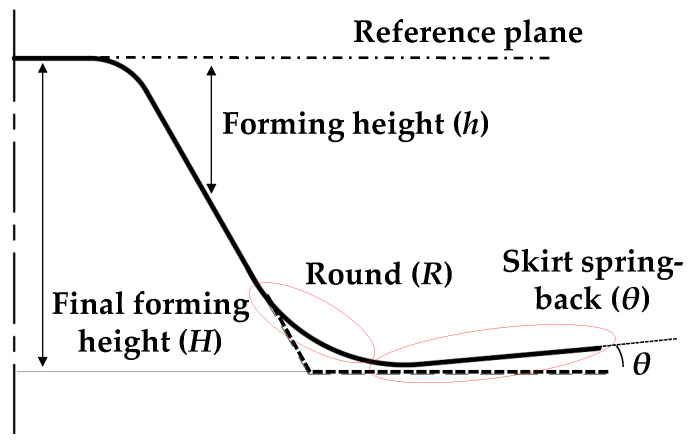
Definition of the shape error.

Figure 4 is a schematic figure of the deformed geometry after the conventional SPIF. The reference geometry is the circular cup shape. The reference plane is the highest surface, which was minimally affected by the incremental forming. The forming height (*h*) denotes the height from the reference plane to the top surface of the sheet. The final forming height (*H*) denotes the forming height from the reference plane to the endpoint. The skirt spring-back (*θ*) denotes the angle between the endpoint and initial contact point in the counter SPIF. The round (*R*) denotes the arc between the slope and the material. The measurement reference point denotes the position of the initial contact point of the tool in the conventional incremental forming process. In this study, an ATOS Core optical 3D scanner with an error of 0.001 mm was used. Additionally, the sheets were scanned on a bed that could rotate 360°. The section profiles of the top surface based on the line connecting the center point of the sheet were compared. 

### 3.2. Tool Path Modification to Increase the Geometric Accuracy 

**Figure 5:**
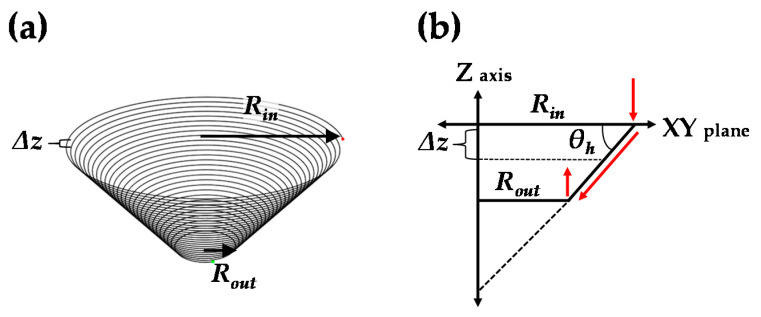
(**a**) Schematic figure of the tool path for circular cup shape and (**b**) sectional view.

First, the tool path of the conventional incremental forming process must be described. The tool path of a circular cup shape is shown in Figure 5. The tool path was controlled by parameters of the entry radius (*R_in_*), exit radius (*R_out_*), forming angle (*θ_h_*), and step-down size (Δ*z*).

**Figure 6:**
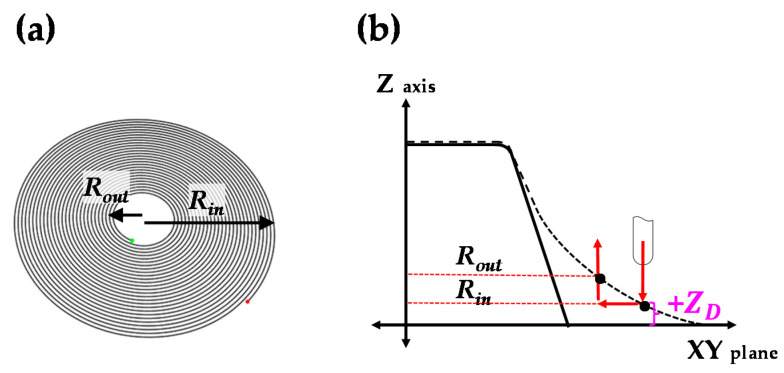
(**a**) Schematic figure of the tool path for the counter SPIF and (**b**) sectional view.

The tool path of counter SPIF was modified according to the shape error. The initial tool path of counter SPIF was disk shaped, as shown in Figure 6. The tool path can decrease the shape error though additional forming of the section deflection region of the circular cup shape. It gives bending deformation in the opposite direction. The tool path is controlled by parameters of the entry radius (*R_in_*), exit radius (*R_out_*), entry depth of tool (*Z_D_*), and step-down size (Δ*z*). The entry depth of the tool (*Z_D_*) is the main process parameter for controlling the bending deformation in the opposite direction. The *Z_D_* is defined with reference to the XY plane. The XY plane denotes the bottom when the sheet is rotated 180° after forming a circular cup shape.

However, the disk-shaped tool path in the counter SPIF results in other shape errors, such as height differences and shape errors according to the angle of the sheet. In the previous work [23], the skirt angle of the product was minimized in counter SPIF by controlling the value of *Z_D_*. However, the height of the incremental formed product was reduced, because the skirt region of the product was deformed in the counter SPIF.

Additionally, the blank in this work was rectangular. The distances between the first contact area of the tool and the blank are different. As a result, the deformed shape of the product is different along one direction. The clover-shaped tool path was adopted to reduce shape error along that direction.

### 3.3. Compensation Process of the Tool Path for the Counter SPIF

**Figure 7:**
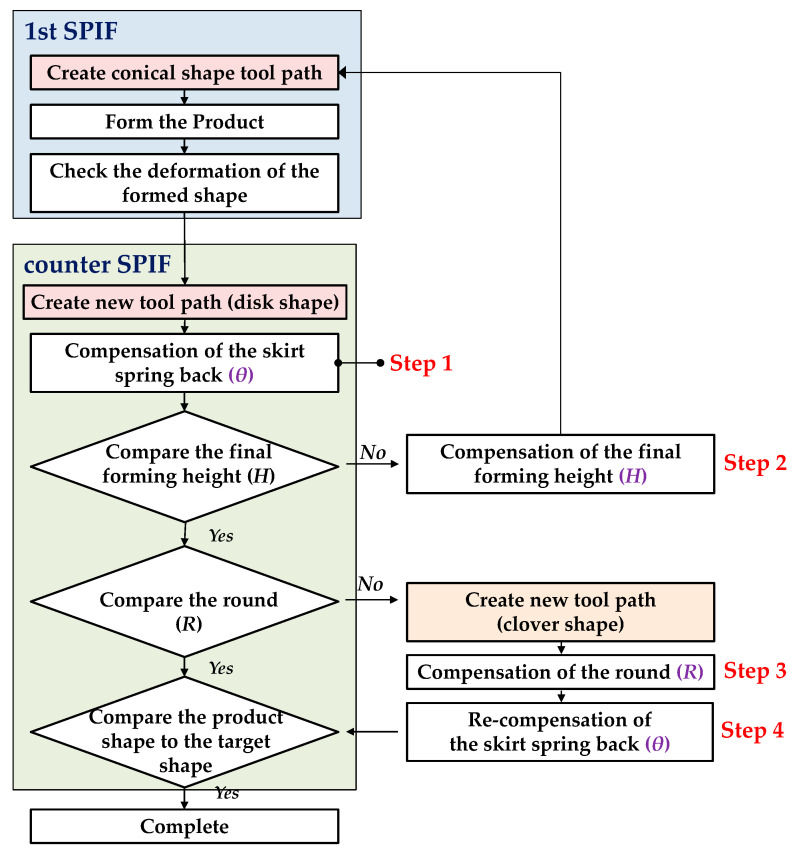
Flow chart to generate the tool path for the two-stage forming process.

Figure 7 shows a flow chart to generate the tool path for the two-stage forming process. In the 1st SPIF, the tool path of the first trial was the same as the tool path in the conventional SPIF [23]. In the counter SPIF, a four-step compensation method was used to decrease the shape error.

In step 1, *θ* was compensated. *Z_D_* in the counter SPIF was determined through the simple optimization method. In step 2, *H* was compensated. Due to the unbending in the counter SPIF, the final forming height of the product decreased. In order to compensate for the final forming height of the circular cup, the tool path in the 1st SPIF was modified. In step 3, *R* was compensated using the changed tool path. When the cross-section showed different results, the tool path of the counter SPIF was modified with the clover shape. In step 4, *θ* was recompensated from the changed tool path, because the modified tool path in step 3 resulted in different sectional geometry. Through the simple optimization methods, the optimized *Z_D_* in the counter SPIF was determined. 

When the difference in geometry by direction was not significant, the optimization could be finished after step 2. 

### 3.4. Experimental Conditions and Measurement Method

In the experiment, incremental forming was performed to decrease the shape error of the circular cup shape. To proceed with the experiment, a sequential forming process was performed. Table 1 lists the initial experimental conditions of the 1st and counter SPIF.

The Δ*z* in the 1st SPIF was 0.3 mm. Δ*z* affects the forming time of the material and surface quality. Therefore, values were selected that would not cause a shallow pattern on the surface of the shape. The Δ*z* in the counter SPIF was zero. That means that the tool moves along the XY plane when the tool contacts the sheet.

The *R_out_* in the counter SPIF was 39 mm because of the diameter of the tool that was considered in the *R_in_* of the 1st SPIF process. The *R_out_* in the counter SPIF was larger than the *R_in_* in the 1st SPIF.

With the process variables in Table 1, it took nearly 7 min for the 1st SPIF process, and it took nearly 4 min for the counter SPIF.

## 4. Experiments of Incremental Forming to Reduce Shape Error

### 4.1. Experimental Results of the 1st SPIF

**Figure 8:**
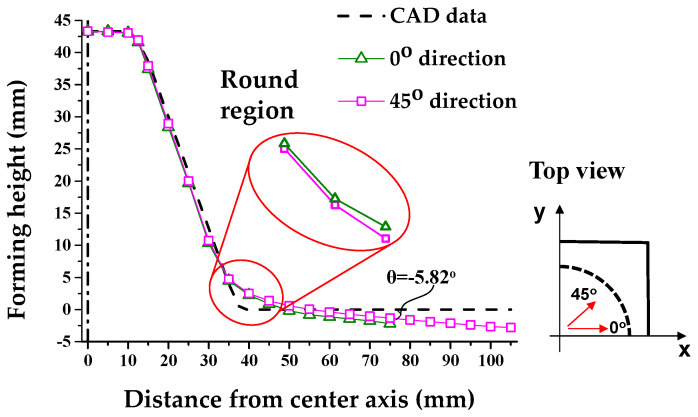
Comparison of the cross-sectional shape after 1st SPIF.

Figure 8 shows the cross-section after the 1st SPIF. The skirt spring-back (*θ*) was measured along the 0° direction. *θ* corresponds to −5.82°, which is different from the CAD data. Thus, it was used to compensate for the *θ* of the skirt to the polynomial expressed as *θ* and *Z_D_*. Furthermore, the forming region to decrease the shape error in the counter SPIF ranged between 45 and 39 mm from the center axis. If the region was formed, the position of the endpoint exceeded the CAD data, given that the position of the skirt was higher than the CAD data. Therefore, compensation for *H* was necessary. 

Additionally, Figure 8 denotes the cross-sectional shape based on the 0° and 45° directions after the 1st SPIF process. Differences in round occur due to the asymmetric shape of the holder. Therefore, we compared 0° and 45°, where the difference for the round occurred the most. Due to the asymmetric geometry of the holder, a difference in *R* values occurred; *R*_0_ corresponded to 33.18 mm and *R*_45_ corresponded to 27.19 mm. Those correspond to the difference in shape error.

### 4.2. Compensation Results in the Counter SPIF

#### 4.2.1. Step 1—Compensation of the Skirt Spring-Back (*θ*)

First, the *Z_D_* was optimized to compensate for the skirt spring-back. The spring-back due to the elastic recovery of the sheet is difficult to predict by simulation or theory. Therefore, it is efficient to optimize *Z_D_* by deriving the polynomial through several experiments. For this reason, the value of *Z_D_* was tested from 1.5 to 1.8 mm at 0.1 mm intervals.

**Figure 9:**
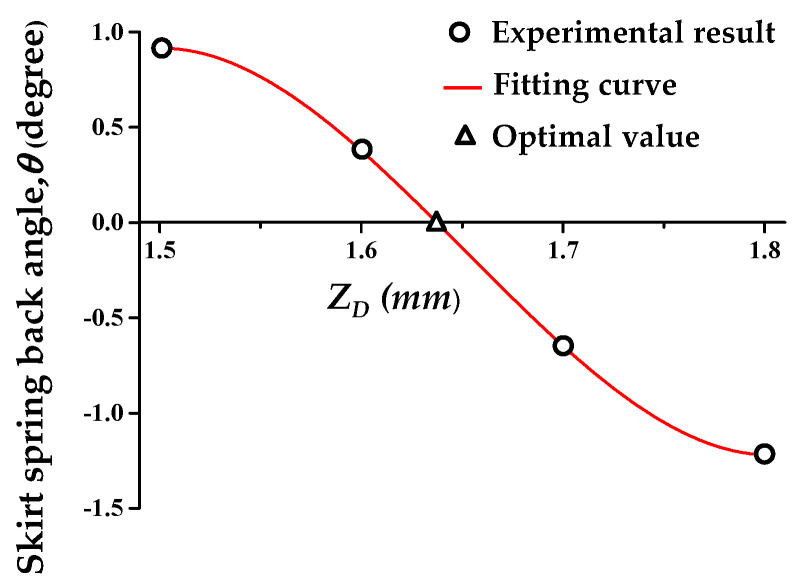
Optimal *Z_D_* for compensation of skirt spring-back.

**Figure 10:**
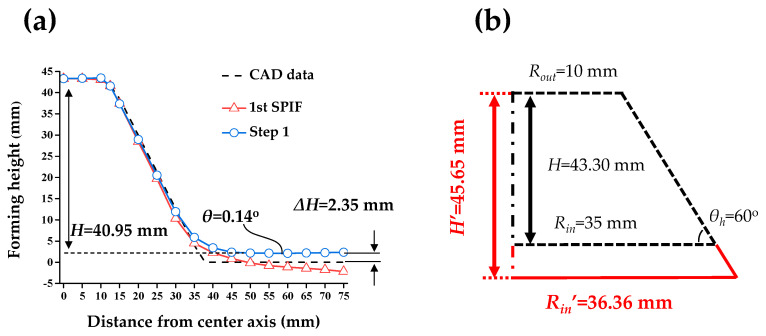
(**a**) Comparison of the cross-sectional shape of the CAD data, 1st SPIF and counter SPIF. (**b**) Modified geometry in the 1st SPIF.

Figure 9 presents the relationship between *Z_D_* and corresponding *θ*. The relationship between *θ* and *Z_D_* was approximated with the 3rd order polynomial presented in Equation (1). *Z_D_* was predicted as 1.63 mm for *θ* corresponding to 0°. The experimental result is shown in Figure 10a. The skirt spring-back angle (*θ*) was 0.14°. When *Z_D_* was smaller than 1.63 mm, *θ* showed a negative value. It means that the counter forming in the counter SPIF occurred excessively.

(1) $$\theta =156.33Z_{D}{}^{3}-775.7Z_{D}{}^{2}+1270.7Z_{D}-688.1$$

#### 4.2.2. Step 2—Compensation of the Final Forming Height (*H*)

After that, the final forming height (*H*) was compensated. After the counter SPIF with the *Z_D_* of 1.63 mm, the height of the circular cup shape was reduced. To solve the problem, the tool path in the 1st SPIF should be modified. The initial tool path (*R_in_* = 35 mm) resulted in *H* of 40.95 mm after the counter SPIF with the *Z_D_* of 1.63 mm. The height of the CAD data in the initial tool path was 43.30 mm. The difference between the CAD data and the experimental result was 2.35 mm. Therefore, it was necessary to newly form a circular cup shape having a final forming height (*H*) of 45.65 mm by adding the difference to the CAD data. *R_in_* was compensated with a value of 36.36 mm. Figure 10b shows a schematic for compensating the final forming height. 

#### 4.2.3. Step 3—Compensation of the Round (*R*)

**Figure 11:**
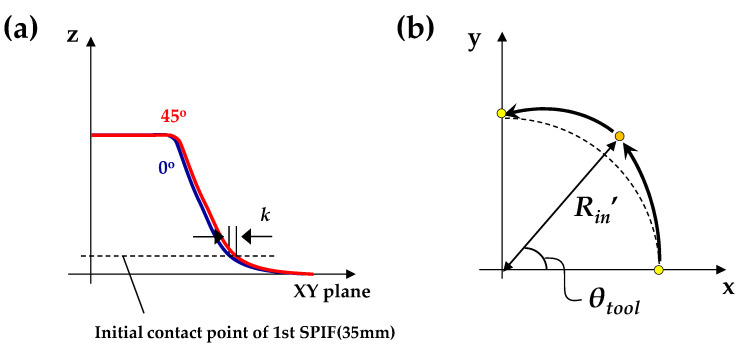
Schematic figure of (**a**) the sectional view along 0° and 45°; (**b**) modified tool path in the counter SPIF for compensation of the round.

*R* was compensated in step 3. A clover-shaped tool path was used as opposed to a disk-shaped tool path to solve the problem. The clover-shaped tool path corresponds to a tool path modified by the difference (*k*) in the radius of the 0° direction (*R*_0_) and the 45° direction (*R*_45_) at the initial contact point in the 1st SPIF process, as shown in Figure 11a. The value of k was defined using Equation (2). The radius of the tool path was modified, as presented in Equation (3). Figure 11b shows the schematic figure of the modified tool path in the counter SPIF.

(2) $$\left(Radius\;of\;45^{{}^{\circ}}direction\left(R_{45}\right)-\left(Radius\;of\;0^{{}^{\circ}}direction\left(R_{0}\right)\right)\right)=k$$

(3) $$R_{in}\prime =R_{in}\times \left(1+k\times \sin{\left(2\theta \right)}^{2}\right)$$

#### 4.2.4. Step 4—Recompensation of the Skirt Spring-Back (*θ*)

Differences in the forming region occurred in the counter SPIF due to the tool path change from disk shaped to clover shaped. To solve the problem, *Z_D_* was optimized once more with the modified tool path of the counter SPIF. Five experiments with different values of *Z_D_* were performed, as shown in Table 2, to express the polynomial in terms of *θ* and *Z_D_* of the tool. The 3rd order polynomial shown in Equation (4) was used, and *Z_D_* was predicted as 1.25 mm when *θ* corresponded to 0°.

(4) $$\theta =1.3917Z_{D}{}^{3}-10.574Z_{D}{}^{2}+14.304Z_{D}-4.0755$$

### 4.3. Experimental Results of the Proposed Method

Table 3 shows the final experimental conditions of the 1st and the counter SPIF processes. These experimental conditions can decrease the shape error of a circular cup shape. 

**Figure 12:**
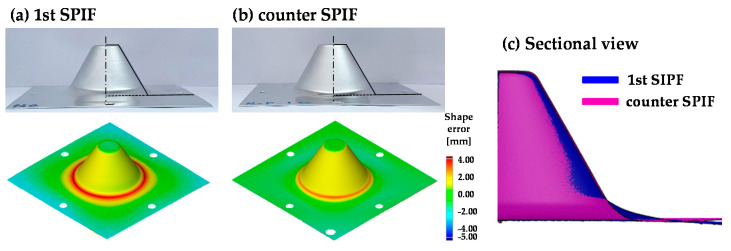
Experimental results and 3D scanned results of the circular cup shape after (**a**) the 1st. and (**b**) the counter SPIF, and (**c**) section view.

Figure 12 shows the experimental results and 3D-scanned data of the circular cup shape of the 1st and the optimized counter SPIF process. The shape error (δ) means the horizontal distance between the CAD data and the experimental results at the measurement reference point. The measurement reference point is the position where the tool initially made contact in the 1st SPIF. The mean value of shape error (δ*_m_*) is the average of the vertical distance between the CAD data and the experimental results. The shape error decreased in the region of the section deflection and skirt after the counter SPIF process. Table 4 shows δ and δ*_m_*. When compared to the 1st SPIF, δ decreased by 67.30% and δ*_m_* decreased by 37.57% after the counter SPIF process. Therefore, it can be concluded that the proposed counter incremental forming process increases shape accuracy.

**Figure 13:**
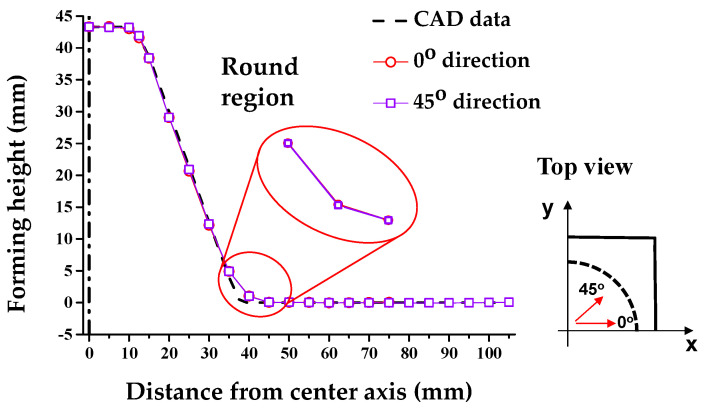
Comparison of the cross-sectional shape after the counter SPIF according to the direction.

Figure 13 shows the cross-sectional after the counter SPIF. The cross-section was extracted from the 3D scanned results. The shape is a rolling direction of a sheet. Thus, the forming accuracy improved when H corresponded to 43.35 mm, and *θ* corresponded to 0.06° only for the 1st SPIF process. Furthermore, Figure 13 shows a cross-sectional shape based on the direction in the combined incremental forming process. Thus, *R* compensated with *R*_0_ corresponded to 12.49 mm and *R*_45_ corresponded to 12.12 mm.

### 4.4. Experimental Results of the Conventional and Proposed Methods

**Figure 14:**
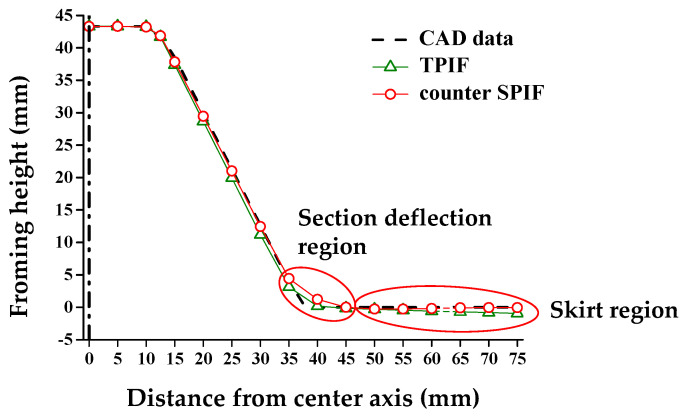
Comparison of the cross-sectional shape in TPIF and counter SPIF.

Two-point incremental forming (TPIF) is known as a conventional incremental forming process to reduce shape errors. In TPIF, the full die, which has the same geometry as the target shape, was employed. Figure 14 is a comparison of the cross-sectional shape between TPIF and the counter SPIF. In the section deflection region, TPIF was more effective than the counter SPIF. The reason was that the radius of the tool was 4 mm, so the range that could be pressed in section deflection region was limited. However, in the skirt region, the counter SPIF, which included the compensation of skirt spring-back, was more consistent with the target shape than TPIF.

### 4.5. Discussion: Cross-Sectional Shape According to the Compensation Steps

**Figure 15:**
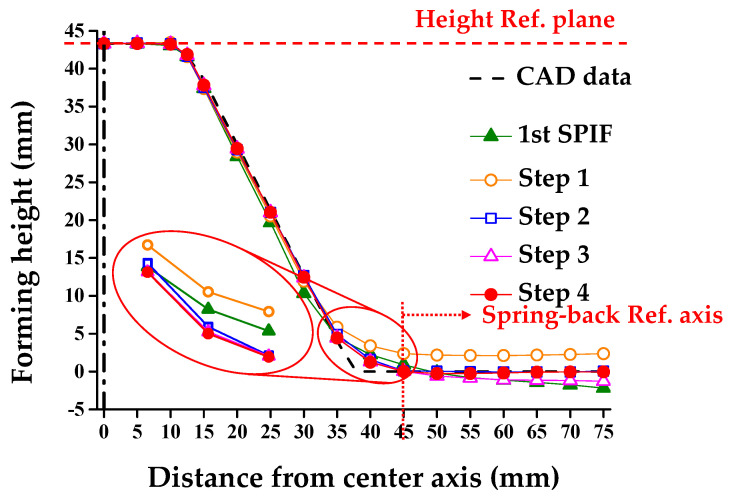
Variation of cross-sectional shape according to the compensation step.

Figure 15 shows a comparison of the cross-sectional shapes from step 1 to step 4 to compensate for the shape error of the circular cup shape in the combined incremental forming process. Table 5 shows the results of the cross-sectional shape. A difference exists in *H*, *θ*, and *R* between the CAD data and the 1st SPIF. 

In step 1, the optimal *Z_D_* through the 3rd order polynomial was derived to compensate for *θ*. As a result, the skirt spring-back was decreased from −5.82° to 0.14°. However, there was a difference between the final forming height. We compensated the initial tool path for the 1st SPIF in step 2. The difference in the forming height from step 1 was added to the CAD data. After that, a clover tool path was used, as opposed to the disk tool path, to compensate for *R* in step 3. Finally, the skirt spring-back recompensation due to tool path change was performed in step 4.

Finally, the accurate geometry was fabricated through the two-stage incremental forming process. The final forming height and the skirt spring-back were optimized. However, due to the limitation of SPIF, the round of the circular cup shape was not minimized. The first round after the 1st SPIF is a common disadvantage of the SPIF process. Since there is no part to hold the material by, unnecessary bending deformation occurs. Even if the counter SPIF is applied, it cannot be reduced perfectly. In order to reduce unwanted bending deformation, TPIF of DSIF process is required.

The tool path compensation technique can be applied to other geometries and materials. Since the final forming height difference may differ depending on the material, the experiments of 1st SPIF are necessary, and the shape can be corrected by utilizing this. Finite element analysis can reduce the required experiments, and research on this is underway.

## 5. Application

### 5.1. Target Geometry

**Figure 16:**
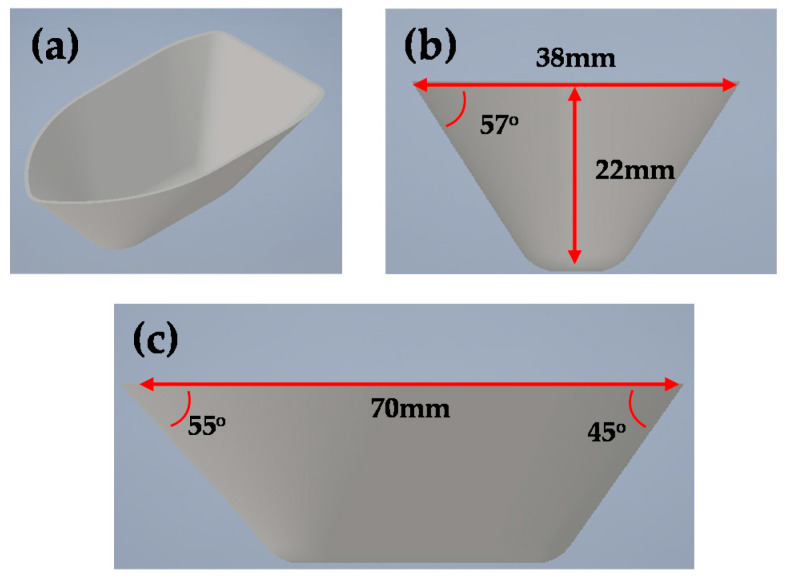
CAD design of the ship-hull shape with (**a**) isometric, (**b**) front, and (**c**) side views.

In the experiment, the ship-hull shape was selected as the target geometry. The test geometry was modified briefly to verify the effect of the proposed counter forming process. Figure 16 shows the schematic of the hull shape. The target geometry is not an axis-symmetric geometry. It has no meaning to compare the geometry with different directions such as 0° and 90°. Therefore, the forming process was modified to step 2.

### 5.2. Experimental Results

**Figure 17:**
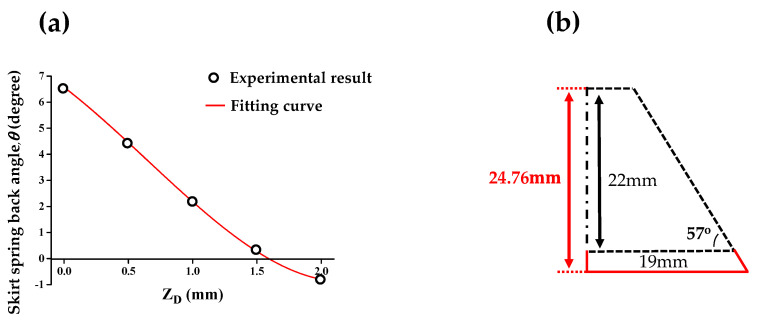
Compensation result of (**a**) the skirt spring-back and (**b**) the final forming height.

First, the 1st SPIF was conducted. After that, the counter SPIF was conducted with different *Z_D_*. Figure 17a presents the distribution of the skirt spring-back angle with respect to *Z_D_*. The relationship was fitted using a 3rd order polynomial, as shown in Equation (5). The value of *Z_D_* resulting in *θ* being zero was 1.59 mm. Finally, the forming height of the product was compensated. The final forming height was compensated in step 2. The height of the 1st tool path was 22 mm. The difference between the CAD geometry and the height of the product after the counter SPIF was 2.76 mm. *H* in the 1st SPIF was compensated to 24.76 mm, as shown in Figure 17b. Due to geometry of the hull plate is an asymmetric shape, the round of the product is not considered.

(5) $$\theta =0.658Z_{D}{}^{3}-1.2675Z_{D}{}^{2}-3.7815Z_{D}+6.57250$$

**Figure 18:**
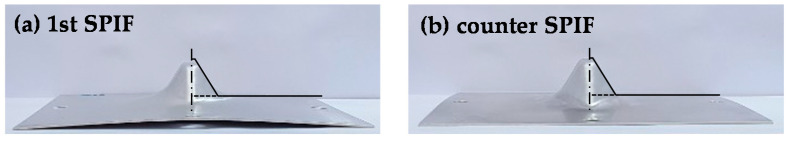
Experimental results of the ship-hull shape after (**a**) the 1st and (**b**) the counter SPIF processes.

**Figure 19:**
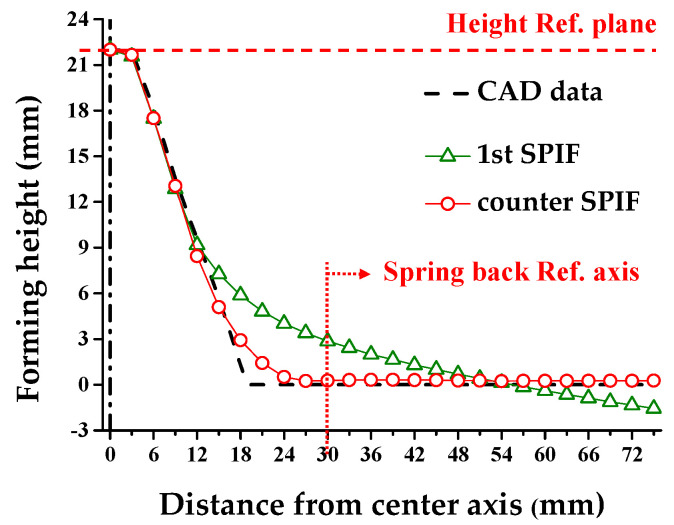
Comparison of the cross-sectional shape in the 1st and the counter SPIF processes.

Figure 18 shows the experimental results of the ship-hull shape after the 1st and the counter SPIF processes. The hull shape exhibited a more reduction effect, as opposed to the circular cup shape. Figure 19 shows a variation in the cross-sectional shape after the 1st SPIF and the counter SPIF. Table 6 shows the comparison results of the cross-sectional shape. The results after the counter SPIF showed more precise geometry.

**Figure 20:**
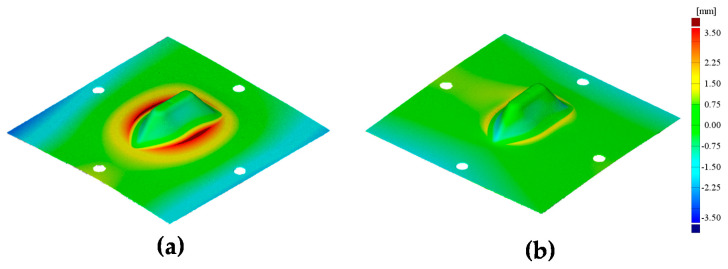
Results of 3D scanned results of the ship-hull shape after (**a**) the 1st and (**b**) the counter SPIF processes.

Figure 20 shows the shape error of the 1st SPIF and the counter SPIF processes using 3D-scanned data. Table 6 shows the δ and δ*_m_*. When compared to the 1st SPIF process, δ decreased by 78.14% and the mean value of δ*_m_* decreased by 40.28% in the counter SPIF. Thus, this demonstrated the reduction in the shape error of the counter SPIF process.

## 6. Conclusions

In the study, the two-stage incremental sheet forming process, including the 1st and the counter SPIF processes, was studied to decrease the shape error. The overall procedure of tool path generation for the two-stage SPIF was developed. Two geometries, a circular cup shape and a ship-hull shape, were manufactured using the developed process. The results of the study are as follows:

(1) The tool path of the proposed counter SPIF was disk-shaped. The main process variable in the counter SPIF was the entry depth of the tool (*Z_D_*). From experiments and the simple optimization methods, optimal *Z_D_* was obtained. It was found that the counter SPIF decreases the skirt spring-back angle effectively. However, the height of the product decreased.
(2) To increase the geometric accuracy, the tool path in the 1st and the counter SPIF was compensated with the 4-step compensation process. The skirt spring-back, section deflection, final forming height, and round were compensated. Through the developed tool paths, the skirt spring-back angle was decreased to 0.05° in the cup shape manufacturing process. At the same time, the section deflection (δ) decreased by 67.30%, and the mean value of shape error (δ*_m_*) decreased by 37.57%.
(3) For another example, the ship-hull shape was adopted. The section deflection (δ) decreased by 78.14%, and the mean value of shape error (δ*_m_*) decreased by 40.28% in the counter incremental forming process compared to the first incremental forming process.
(4) It was found that the proposed two-stage SPIF was very effective at increasing the geometric accuracy. The two-stage SPIF does not require additional experimental devices such as a die-set or robot arm. The two-stage SPIF is expected to be helpful in the sheet metal forming industry, because it improves geometric accuracy and reduces manufacturing equipment cost.

## Figures and Tables

**Table 1:** Initial experimental conditions of the 1st and the counter SPIF.

Stage	Tool Path Shape	*R_in_* (mm)	*R_out_* (mm)	*θ_h_* (degree)	∆*z* (mm)
1st SPIF	circular cup	35	10	60	0.3
counter SPIF	disk	45	39	-	0

**Table 2:** Skirt spring-back of the circular cup shape based on *Z_D_* in the step 4.

***Z_D_* (mm)**	1.1	1.2	1.3	1.4	1.5
***θ* (degree)**	0.72	0.28	−0.33	−0.95	−1.71

**Table 3:** Final experimental conditions of (a) the 1st and (b) the counter SPIF.

SPIF	Tool Path Shape	*R_in_* (mm)	*R_out_* (mm)	*θ_h_* (°)	∆*z* (mm)	*Z_D_* (mm)
(a) 1st SPIF	circular cup	36.36	10	60	0.3	-
(b) counter SPIF	clover (according to step 3)	46.36	40.36	-	0.3	1.25

**Table 4:** Initial experimental conditions of the 1st and the counter SPIF.

Shape Error	CAD Data	1st SPIF	Counter SPIF
δ (mm)	0	3.15	1.03
δ*_m_*(mm)	0	1.73	1.08

**Table 5:** Comparison of the circular cup shape according to each compensation step.

Parameter	CAD Data	1st SPIF	Counter SPIF			
			Step 1	Step 2	Step 3	Step 4
*θ* (degree)	0	−5.82	0.14	0.11	−2.57	0.05
*H* (mm)	−43.30	−45.49	−40.95	−43.25	−44.58	−43.35
*R*_0_/*R*_45_ (mm)	-	33.18/27.19	21.18/15.68	19.08/13.65	17.81/17.79	12.49/12.12

**Table 6:** Comparison of hull shape according to each process.

Parameter	CAD Data	1st SPIF	Counter SPIF	
			Step 1	Step 2
*θ* (degree)	0	−5.61	0.08	0.03
*H* (mm)	−22	−23.56	−19.24	21.73
δ (mm)	0	3.75	3.75	0.82
δ*_m_*(mm)	0	1.44	1.44	0.86
